# Optimizing the Dopant and Carrier Concentration of Ca_5_Al_2_Sb_6_ for High Thermoelectric Efficiency

**DOI:** 10.1038/srep29550

**Published:** 2016-07-13

**Authors:** Yuli Yan, Guangbiao Zhang, Chao Wang, Chengxiao Peng, Peihong Zhang, Yuanxu Wang, Wei Ren

**Affiliations:** 1 Institute for Computational Materials Science, School of Physics and Electronics, Henan University, Kaifeng 75004, People’s Republic of China; 2 Department of Physics, International Center for Quantum and Molecular Structures, and Materials Genome Institute, Shanghai University, Shanghai 200444, China; 3 Department of Physics, University at Buffalo, State University of New York, Buffalo, New York 14260, USA

## Abstract

The effects of doping on the transport properties of Ca_5_Al_2_Sb_6_ are investigated using first-principles electronic structure methods and Boltzmann transport theory. The calculated results show that a maximum *ZT* value of 1.45 is achieved with an optimum carrier concentration at 1000 K. However, experimental studies have shown that the maximum *ZT* value is no more than 1 at 1000 K. By comparing the calculated Seebeck coefficient with experimental values, we find that the low dopant solubility in this material is not conductive to achieve the optimum carrier concentration, leading a smaller experimental value of the maximum *ZT*. Interestingly, the calculated dopant formation energies suggest that optimum carrier concentrations can be achieved when the dopants and Sb atoms have similar electronic configurations. Therefore, it might be possible to achieve a maximum *ZT* value of 1.45 at 1000 K with suitable dopants. These results provide a valuable theoretical guidance for the synthesis of high-performance bulk thermoelectric materials through dopants optimization.

The global trend of energy use is moving towards sustainable development and the waste-to-energy concept is being highly promoted as a part of this effort^1^. Thermoelectric devices can convert some of waste heat into useful electricity. In essence, thermoelectric coolers and generators are heat engines thermodynamically similar to conventional vapor power generation or heat pumping cycles, but they use electrons as the working fluid instead of gases or liquids^2^. Thus, thermoelectric devices have the advantages of being solid-state devices, low maintenance costs and long lifetime^3^. Currently, the two main focuses in thermoelectrics research are the discovery of new materials with high thermoelectric efficiency^4^,^5^,^6^,^7^,^8^ and the design and optimization of thermoelectric generators^9^,^10^,^11^,^12^. Our work focuses on materials optimization for thermoelectric applications.

The thermoelectric efficiency of a material is governed by its thermoelectric figure of merit *ZT* = *S*^2^*σT*/*κ*, where *S* is the Seebeck coefficient or thermopower, *σ* is the electrical conductivity, *T* is temperature, and *κ* is the thermal conductivity. The thermal conductivity has both the electric and lattice contributions: *κ* = *κ*_*e*_ + *κ*_*l*_. The figure of merit formula implies that a material suitable for thermoelectric applications must have a large *S*, high *σ*, and low *κ*. However, increasing *σ* by increasing the carrier concentration usually leads to a decrease in the magnitude of *S* and an increase in *κ*_*e*_. The *κ*_*l*_ is generally considered to be the most uncoupled property in the expression of *ZT*, thus may be tuned independently.

Ca_5_Al_2_Sb_6_ is a promising thermoelectric material, not only because Ca, Al, and Sb are inexpensive and nontoxic but also because Ca_5_Al_2_Sb_6_ possesses an extremely low lattice thermal conductivity. In addition, the total *κ* is not significantly affected by doping^13^,^14^,^15^. More importantly, Ca_5_Al_2_Sb_6_ contains both covalent and ionic bondings, leading to a fairly complex crystal structure as shown in Fig. 1. A 2 × 2 × 1 supercell is shown in the figure for a better illustration of the bonding structure. This complex structure provides ample space for materials optimization, and controlling the carrier concentration has been a primary means to improve the thermoelectric conversion efficiency (*ZT*) of Ca_5_Al_2_Sb_6_. Several experiments have been done to improve the thermoelectric properties of Ca_5_Al_2_Sb_6_ by tuning the carrier concentration^13^,^14^,^15^,^16^. For example, Na^1+^ doping on the Ca^2+^ sites results in the highest figure of merit (a peak *ZT* of 0.6 at 1000 K)^13^. It is known that Na has disadvantage of having a low solubility and low doping effectiveness, and Zn^2+^ (substituting Al^3+^) is a more effective dopant. Surprisingly, the *ZT* values of Zn doped samples are lower than those of Na doped samples^14^. Incomplete dopant activations yielding low hole concentrations have been observed in Mn^2+^ doped sample (substituting the Al^3+^ sites) and higher *ZT* values have not been achieved via Mn doping^15^.

There have also been a few theoretical studies reported for the thermoelectric properties of Ca_5_Al_2_Sb_6_^17^,^18^,^19^. For example, in an earlier paper^17^, we have employed first-principles calculations and Boltzmann transport theory to investigate the thermoelectric performance of Ca_5_Al_2_Sb_6_, and have obtained results in terms of the thermoelectric powerfactor to the relaxation time ratio (*S*^2^*σ*/*τ*)^17^. In order to better compare with experiments and provide a theoretical guidance for future experiments, in this work, we use the method of Ong and coworkes^20^ to eliminate the uncertainly in the relaxation time *τ* and estimate the values of *ZT* using the experimental lattice thermal conductivity. The calculated results show that the maximum *ZT* value at optimum carrier concentration is 1.45 at 1000 K. However, experimental studies have indicated that the maximum *ZT* value was no more than 1^13^,^14^,^15^. In this paper, we would like to address the discrepancy between theory and experiment and provide some general guidance for future materials optimization towards achieving a maximum *ZT* value.

## Result and Discussion

### Band Structure

Our calculations using the TB-mBJ^21^ potential result a nearly direct band gap of about 0.52 eV (shown in Fig. 2), which agrees well with the experimental value of 0.5 eV^13^. This suggests that the TB-mBJ method may give more reliable results compared with a previous study using the Perdew-Burke-Ernzerhof (PBE) functional^17^. High thermoelectric performance is generally found in heavily-doped semiconductor with carrier concentrations on the order of 10^19^ to 10^21^ cm^−3^ 22. Therefore, we focus on understanding the properties of heavily doped Ca_5_Al_2_Sb_6_. For metals or degenerate semiconductors, the Seebeck coefficient (*S*) is given by^22^


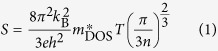


where *k*_B_ is Boltzmann’s constant, *e* the electron charge, *n* the carrier concentration, and 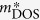 the density-of-state effective mass. 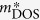 = 
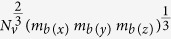
 for an anisotropic material having band masses of *m*_*b*(*x*)_, *m*_*b*(*y*)_, and *m*_*b*(*z*)_ along the three principle directions. *N*_*v*_ is the degeneracy of the valence states near the Fermi level. Equation (1) shows *S* is proportional to 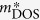, temperature *T*, and 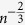. On the other hand, from Table 1, we can see that 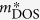 of p-type doping is greater than that of n-type doping. Therefore, given the same temperature and carrier concentration, p-type materials should have higher Seebeck coefficients than those of n-type materials. The electrical conductivity is given by


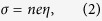


where *η* is the carrier mobility. It is well known that the mobility *η* is inversely proportional to inertial mass *m*_I_ [*m*_I_ = 3/(1/*m*_*b*(*x*)_ + 1/*m*_*b*(*x*)_ + 1/*m*_*b*(*x*)_)]. Table 1 also shows that the inertial mass *m*_I_ of p-type doping is smaller than that of n-type doping. So for the same carrier concentration and temperature, *σ* of the p-type material should be larger than that of the n-type.

### Transport properties of Ca_5_Al_2_Sb_6_

The synthesized Ca_5_Al_2_Sb_6_ samples are polycrystalline^13^,^14^,^15^,^23^. The scattering from grain boundaries will affect the transport properties. There is experimental evidence, however, that the boundary effects become less important with increasing temperature. For example, Atakulov *et al*.^24^ found that for the same electron concentration, the effect of grain boundary scattering on the electron mobility is negligible when the temperature reaches 400 K. Snyder’s team^13^,^14^,^15^,^23^ also reported that the polycrystalline samples have minor effect on the carrier mobility of Ca_5_Al_2_Sb_6_ doped with Na, Zn, and Mn above 300 K. Therefore, at high temperatures, the most important effects of the polycrystalline samples are the random orientation of the grains. In this work, the transport properties of Ca_5_Al_2_Sb_6_ are calculated by averaging over three principal crystal axes. The anisotropic transport properties are shown in supplemental Fig. 1. Taking into account the solubilities of dopants in Ca_5_Al_2_Sb_6_, we only report the calculated transport coefficient for carrier concentration ranging from −0.4 *e*/*uc* to −0.01 *e*/*uc* and 0.01 *h*/*uc* to 0.4 *h*/*uc*. These parameters are more realistic than those used in our previous work (from −6.5 *e*/*uc* to 6.5 *h*/*uc*)^17^.

Figure 3(a,b) show the calculated Seebeck coefficient as a function of the carrier concentration for n-type and p-type Ca_5_Al_2_Sb_6_ for *T* = 300, 500, 800, 1000, and 1200 K. Comparing the two figures, we can see that, regardless of n-type or p-type doping, the absolute values of *S* increase with increasing temperature and decreasing carrier concentration between 300 K and 800 K. At the same temperature and carrier concentration, the absolute values of *S* for the p-type systems are larger than those of n-type ones. These results are consistent with the understanding that 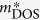 (hole) > 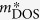 (electron). However, between 1000 K and 1200 K, the absolute value of *S* first increases with increasing carrier concentration and decreasing temperature, then decreases with increasing carrier concentration. This is likely due to the enhanced bipolar transport effects at high temperature.

We have also carried out calculations beyond the rigid-band approximation by introducing dopants to the system explicitly as shown in Supplemental Fig. 3. In this calculation, one Sb is substituted by one Ga, which naturally introduces hole carriers in the system. Additional carriers can be further introduced to the system starting from the band structure calculated with dopants included. We find that the overall trend of the calculated transport properties remains unchanged. The calculated optimal *ZT* values, however, are slightly lower than those obtained using the rigid-band approximation due to presence of defect states and distortions to the band structure near the Fermi level. Unfortunately, realistic modeling of effects of impurities is still challenging due to the enormous computational cost.

As will be discussed later, the hole carrier concentration for the Ca_4.75_Na_0.25_Al_2_Sb_6_ system is about 0.07 *h*/*uc* at 800 K^13^. At this temperature, the reported experimental resistivity is approximately 8.0 × 10^−5^ Ω ⋅ m, which can be combined with the calculated *σ*/*τ* to give a relaxation time *τ* = 1.7 × 10^−15^ s for Ca_4.75_Na_0.25_Al_2_Sb_6_ at 800 K. Near this temperature, the experimental data for this sample and others follow an approximate electron–phonon *T* dependence, i.e., 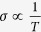. Taking into account the doping effects, we have *τ* = 8.68 × 10^−6^* T*^−1^*n*^−1/3^. Figure 3(c,d) show *σ* as a function of carrier concentration for n-type and p-type Ca_5_Al_2_Sb_6_ for *T* = 300, 500, 800, 1000, and 1200 K. Regardless of n-type or p-type doping, the electrical conductivities increase with increasing carrier concentration. These figures also show that for the same carrier concentration, the conductivity decreases with increasing temperature as a result of decreased carrier mobilities. At the same temperature and carrier concentration, the absolute values of *σ* of p-type doping are larger than those of n-type doping, which can be explained by the fact that *m*_I_ (hole) < *m*_I_ (electron).

Previous experimental studies have shown that Ca_5_Al_2_Sb_6_ possesses an extremely low lattice thermal conductivity (0.6 WmK^−1^ at 850 K), and the total thermal conductivity is not significantly affected by doping^13^,^14^,^15^ (also see supplemental Fig. 2 for more details). Here we use the experimental thermal conductivity of Ca_4.75_Na_0.25_Al_2_Sb_6_ from the work of Toberer *et al*.^13^, which falls within the range of the measured values for similar systems. The calculated *ZT* as a function of *T* and carrier concentration are shown in Fig. 3(e,f). Comparing the two figures, we find that, at the same temperature, the values of *ZT* for the p-type systems are much higher than those for the n-type ones. Interestingly, Ca_5_Al_2_Sb_6_ almost always has excess holes^13^,^14^,^15^,^23^. As depicted in the figure, the optimum carrier concentration changes from −0.01 *e*/*uc* at 300 K to 0.18 *e*/*uc* at 1200 K for electron-doped systems and changes from 0.01 *h*/*uc* at 300 K to 0.17 *h*/*uc* at 1200 K for hole-doped ones. The maximum figure of merit at the optimum carrier concentration increases from 0.21 at 300 K to 0.95 at 1200 K for n-type materials and from 0.24 at 300 K to 1.65 at 1200 K for p-type ones. Our results suggest that the maximum *ZT* value at 1000 K is 1.45. However, experimental studies have indicated that the maximum *ZT* value is no more than 1 at 1000 K^13^,^14^,^15^. This discrepancy between theory and experiment motivates us to look into other factors that may affect the performance of the experimental samples and how one can further improve the experimental *ZT* value of Ca_5_Al_2_Sb_6_.

### Choosing suitable dopants

In order to understand the remarkable difference in the maximum *ZT* value between theory and experiment, a comparison between the calculated and experimental values^13^,^14^,^15^ of the Seebeck coefficient is shown in Fig. 4, where *p* represents the theoretical hole carrier per unit cell. Comparing the calculated and experimental values of *S*, we find that results for the supposedly intrinsic samples^13^,^14^ are to be compared with the theoretical, carrier concentration of about 0.03–0.055 *h*/*uc*. This suggests that unintentional doping cannot be overlooked. In addition, the results for the Ca_4.95_Na_0.05_Al_2_Sb_6_ sample^13^ are better compared with theoretical results calculated with 0.05–0.06 *h*/*uc*, which is only half of the nominal doping. Note that each unit cell contains two Ca_5_Al_2_Sb_6_ formula units. For Ca_5_Al_1.95_Mn_0.05_Sb_6_^15^ and Ca_5_Al_1.95_Zn_0.05_Sb_6_^14^, the corresponding hole densities are approximately 0.065–0.07 *h*/*uc*. Therefore, there is a significant difference between the nominal doping and the carrier concentration, and the solubility limit may play an important role. These results suggest that in the high doping region, the solid solubility limits of Na, Mn, and Zn in Ca_5_Al_2_Sb_6_ may be a determining factor that controls the carrier concentration in experiment. As we have mentioned in the previous section, the optimum carrier concentration changes from 0.01 *h*/*uc* at 300 K to 0.17 *h*/*uc* at 1200 K.

Therefore, while comparing with the experimental results, one must consider the solubility limit of the dopant. In order to achieve the optimal carrier concentration, it is important to find suitable dopants. To this end, we have calculated the formation energy Δ*E* for Ca_5−*x*_M_*x*_Al_2_Sb_6_ (M = Na, Mg, and Ga), Ca_5_Al_2−*x*_M_*x*_Sb_6_ (M = Ga, Mn, and Zn), and Ca_5_Al_2_Sb_6−*x*_M_*x*_ (M = Ge, Ga, and Zn). As an example, the formation energy of Ca_5−*x*_Na_*x*_Al_2_Sb_6_ is defined as


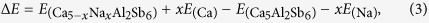


where 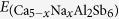 and 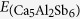 are the total energies of the Ca_5_Al_2_Sb_6_ with and without doping, respectively. *E*_(Ca)_ and *E*_(Na)_ are the total energies per atom of Ca and Na solids, respectively, and *x* is the dopant concentration. We used a 2 × 1 × 2 supercell containing 104 atoms for all formation energy calculations. A comparison of the formation energy for different dopants is given in Table 2. It is important to point out that the calculated formation energies are negative for Ca_5_Al_2−*x*_Ga_*x*_Sb_6_ and Ca_5_Al_2_Sb_6−*x*_Ge_*x*_ (*x* = 0.125, 0.25, and 0.375), which suggests that these doping positions and dopants are energetically favorable. The formation energies are positive for Ca_5−*x*_Na_*x*_Al_2_Sb_6_, Ca_5−*x*_Mg_*x*_Al_2_Sb_6_, Ca_5−*x*_Ga_*x*_Al_2_Sb_6_, Ca_5_Al_2−*x*_Mn_*x*_Sb_6_, and Ca_5_Al_2−*x*_Zn_*x*_Sb_6_ (*x* = 0.125, 0.25, and 0.375), indicating that they are thermodynamically unstable. Interestingly, the formation energies of Ca_5_Al_2_Sb_6−*x*_Ga_*x*_ and Ca_5_Al_2_Sb_6−*x*_Zn_*x*_ change from negative to positive, which suggests a decreasing thermodynamic stability with increasing carrier concentration. From Table 2, we can conclude that Sb is the most favorable site for substation, which is followed by Al, and Ca is least favorable site.

**Figure 1:**
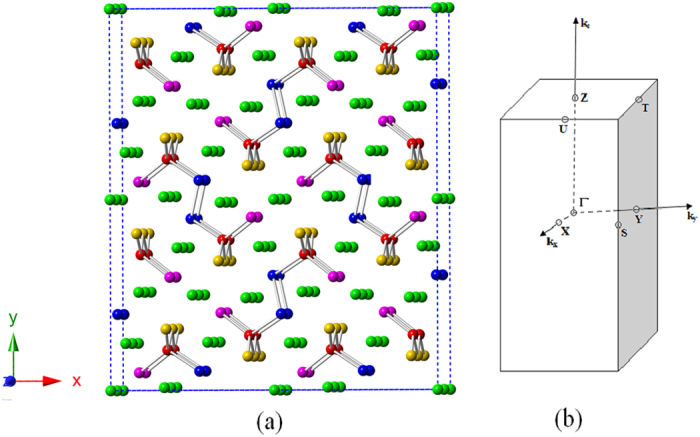
(**a**) Crystal structure of a 2 × 2 × 1 Ca_5_Al_2_Sb_6_ supercell viewed along the z-axis. Green, red, orange, magenta, and blue spheres indicate Ca, Al, Sb(1), Sb(2), and Sb(3) atoms, respectively. (**b**)Brillouin zone of Ca_5_Al_2_Sb_6_.

**Figure 2:**
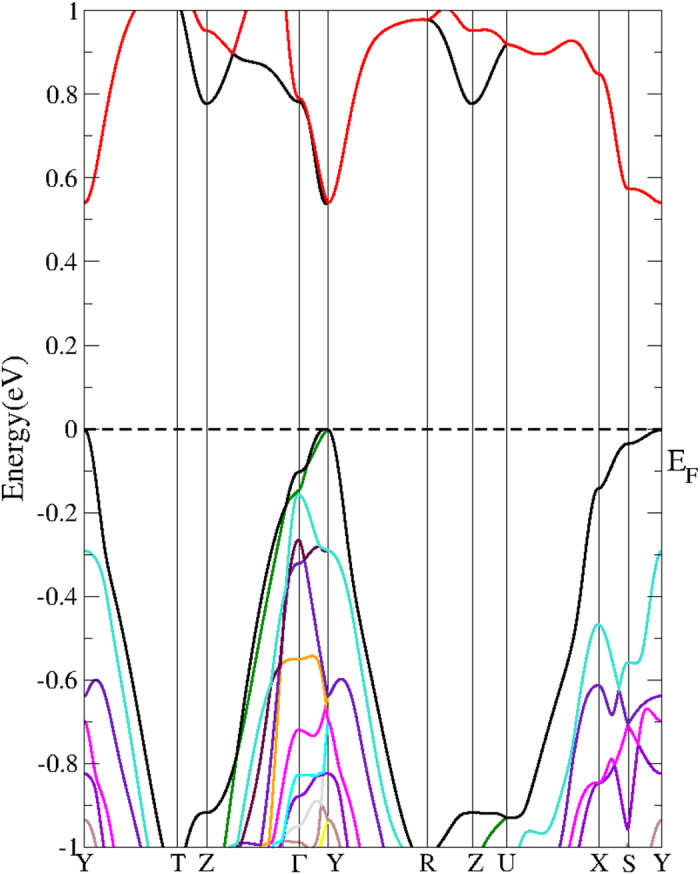
Calculated band structure of Ca_5_Al_2_Sb_6_ using the TB-mBJ potential. The energy zero is at the valence-band maximum.

**Figure 3:**
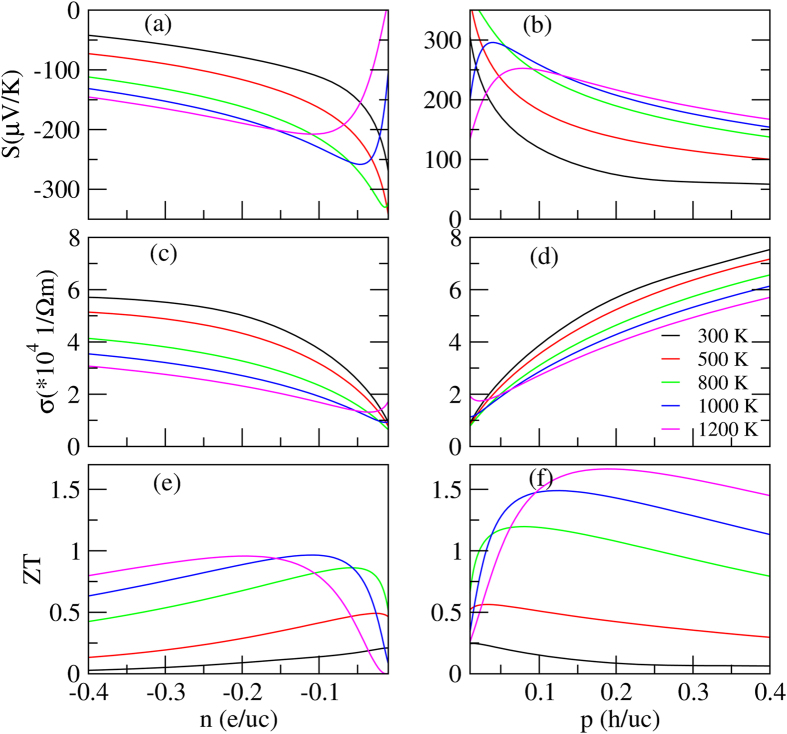
Thermoelectric coefficients as functions of the carrier concentration for n-type (left) and p-type (right) Ca_5_Al_2_Sb_6_ for *T* = 300, 500, 800, 1000, and 1200 K.

**Figure 4:**
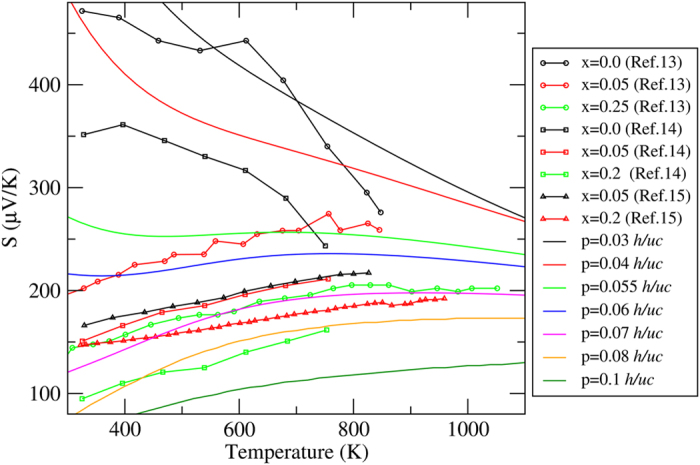
Calculated *S* versus temperature compared with experimental data for Ca_5−*x*_Na_*x*_Al_2_Sb_6_ [Toberer*et al.*^13^], Ca_5_Al_2−*x*_Mn_*x*_Sb_6_ [Zevalkink *et al.*^14^], and Ca_5_Al_2−*x*_Zn_*x*_Sb_6_ [Snyder *et al.*^15^], where *p* presents the theoretical hole carrier concentration.

It would be interesting to understand why Al and Sb positions are suitable doping positions and how one can select appropriate dopants to achieve optimal carrier concentrations. In Ca_5_Al_2_Sb_6_, the anionic building block is [Al_2_Sb_6_]^−10^ and Ca atoms donate all of their valence electrons to the Al_2_Sb_6_ structure. The Sb(3)-Sb(3) and the Al-Sb bonds are weak to moderate covalent bonds, as shown Fig. 1(a). There is a strong Coulomb interaction between Ca cations and the [Al_2_Sb_6_]^−10^ anion. Therefore, in Ca_5_Al_2_Sb_6_, substituting Ca is the most difficult and substitution of Sb(3) is the easiest. This is consistent with the conclusion from our formation energy calculations. We also find that, for a given doping position, the formation energy is closely related to the electronic configuration of the dopant. It appears that dopants with an electronic configuration that is similar to the atom being substituted usually have low formation energies. For example, the valence electronic configuration of Sb is 5*s*^2^5*p*^3^. Therefore, according to the electronic configuration of the dopant, there are two classes of suitable dopants. The first class have partially occupied 4*p* state (e.g., Ge: 4*s*^2^4*p*^2^ and Ga: 4*s*^2^4*p*^1^), which is very similar to that of Sb. The second class have unoccupied 4*p* orbital (e.g., Zn: 4*s*^2^4*p*^0^). Therefore, we conclude that it is easier to dope the system when by substituting Sb atoms with electronically compatible dopants.

## Conclusion

In conclusion, we have investigated the doping effects on the thermoelectric performance of Ca_5_Al_2_Sb_6_ using first-principles electronic structure methods coupled with Boltzmann transport theory. We find that a maximum *ZT* value of 1.45 can be achieved with an optimum carrier concentration at 1000 K. This value is significantly higher than experimental measurement. We point out that the discrepancy between theory and experiment is likely a result of limited solubility of dopants. Our calculations suggest that substituting Sb with electronically compatible dopants may help to reach optimal carrier concentrations, thus achieving the predicted *ZT* limit.

## Computational Detail

The projector augmented wave method of Blöchl^25^ as implemented within the Vienna Ab-initio Simulation Package (VASP)^26^,^27^ is used for structural optimization for the ideal and doped crystal structures. The Perdew-Burke-Ernzerhof-(PBE)generalized gradient approximation^28^ within the density functional theory is used. A plane wave kinetic energy cut-off of 500 eV is used for all calculations. For the Brillouin zone integration, a 5 × 5 × 5 Monkhorst-Pack^29^ *k* point grid is used for the 26-atom primitive cell. We optimize both the lattice constants and atomic positions for all systems studied. Impurity formation energies are calculated with 2 × 1 × 2 supercells containing 104 atoms. Atoms are relaxed until the residual forces are smaller than 0.02 eV/Å. The effects of similar ionic radius substitutions (e.g., Na, Mg, and Ga for Ca; Ga, Zn, and Mn for Al; and Ge, Zn, and Ga for Sb) are studied. We find that the Sb(3) sites are the most energetically favorable.

Since the electronic transport properties are strongly affected by the band-edge states, highly accurate density functional theory calculations are performed with the WIEN2k code^30^ based on the full-potential linearized augmented plane-wave (FLAPW) method. Through a systematic comparison of results calculated using different exchange-correlation potentials^21^,^28^,^31^,^32^,^33^, we find that the band gap calculated with a modified Beck–Johnson (TB-mBJ) potential provides the best agreement with experiments^13^,^23^. Therefore, we use the TB-mBJ potential for electronic structure calculations in this work. In FLAPW calculations, we use R_*MT*_K_max_ = 9, which determines the matrix size, where K_max_ is the plane wave cut-off and R_*MT*_ is the smallest atomic radius. The muffin-tin radii are chosen to be 2.5 a.u. for Ca, Al, and Sb. Self-consistent calculations are carried out with 1500 k points in the irreducible Brillouin zone and the total energy is converged to within 0.0001 Ry. We include the scalar-relativistic effects for Sb. The results from the electronic structure calculations provide the necessary inputs for calculating the transport using the Bolt*ZT*rap code^34^,^35^ under the assumption that the relaxation time *τ* is direction independent.

## Additional Information

**How to cite this article**: Yan, Y. *et al*. Optimizing the Dopant and Carrier Concentration of Ca_5_Al_2_Sb_6_ for High Thermoelectric Efficiency. *Sci. Rep.* **6**, 29550; doi: 10.1038/srep29550 (2016).

## Supplementary Material

Supplementary Information

## Figures and Tables

**Table 1:** Calculated band effective masses along three principle axes, density of states effective mass, and inertial effective mass.

	*m*_*b*(*x*)_	*m*_*b*(*y*)_	*m*_*b*(*z*)_	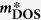	*m*_I_
Electrons	3.23	0.59	1.62	4.810	2.6218
Holes	6.61	2.15	0.54	6.513	1.1564

**Table 2:** Formation Energy (eV) of Ca_5−*x*_M_*x*_Al_2_Sb_6_, Ca_5_Al_2−*x*_M_*x*_Sb_6_ and Ca_5_Al_2_Sb_6−*x*_M_*x*_.

*x*	Ca_5−*x*_M_*x*_Al_2_Sb_6_			Ca_5_Al_2−*x*_M_*x*_Sb_6_			Ca_5_Al_2_Sb_6−*x*_M_*x*_		
	Na	Mg	Ga	Ga	Mn	Zn	Ge	Ga	Zn
0.125	2.943	2.952	3.606	−1.677	0.289	0.255	−1.321	−0.917	−0.710
0.25	3.911	2.524	4.542	−1.569	2.414	0.708	−0.674	0.1633	0.994
0.375	5.571	2.334	5.478	−1.459	4.456	1.208	−0.062	1.290	2.196

## References

[b1] Ng W. P. Q., Lam H. L., Varbanov P. S. & Klemes J. J. Waste-to-Energy (WTE) network synthesis for Municipal Solid Waste (MSW). Energy Convers. Manage 85, 866–874 (2014).

[b2] Heremans J. P., Jovovic V., Toberer E. S., Saramat A. & Kurosaki K. . Enhancement of thermoelectric efficiency in PbTe by distortion of the electronic density of states. Science 321, 554–557 (2008). 18653890 10.1126/science.1159725

[b3] Wijesekara W., Rezania A. & Rosendahl L. Simple engineering design for complex thermoelectric generators based on reduced current approach. Energy 86, 455–466 (2015).

[b4] Bathula S., Jayasimhadri M., Gahtori B., Singh N. K. & Tyagi K. . The role of nanoscale defect features in enhancing the thermoelectric performance of p-type nanostructured SiGe alloys. Nanoscale 7, 12474–12483 (2015). 26138852 10.1039/c5nr01786f

[b5] Sun L., Jiang P. H., Liu H. J., Fan D. D. & Liang J. H. . Graphdiyne: A two-dimensional thermoelectric material with high figure of merit. Carbon 90, 255–259 (2015).

[b6] Zhang J., Liu H. J., Cheng L., Wei J. & Liang J. H. . Phosphorene nanoribbon as a promising candidate for thermoelectric applications. Sci. Rep. 4, 6452 (2014). 25245326 10.1038/srep06452 PMC4171703

[b7] Ihnatsenka S., Crispin X. & Zozoulenko I. V. Understanding hopping transport and thermoelectric properties of conducting polymers. Phys. Rev. B 92, 035201-1–035201-12 (2015).

[b8] Choi W., Jun D., Kim S., Shin M. & Jang M. Thermoelectric characteristics of Pt-silicide/silicon multi-layer structured p-type silicon. Energy 82, 180–183 (2015).

[b9] Kossyvakis D. N., Vossou C. G., Provatidis C. G. & Hristoforou E. V. Computational analysis and performance optimization of a solar thermoelectric generator. Renew. Energy 81, 150–161 (2015).

[b10] Fisac M., Villasevil F. X. & Lopez A. M. Design of a thermoelectric generator with fast transient response. Renew. Energy 81, 658–663 (2015).

[b11] Aranguren P., Astrain D., Rodriguez A. & Martinez A. Experimental investigation of the applicability of a thermoelectric generator to recover waste heat from a combustion chamber. Appl. Energy 152, 121–130 (2015).

[b12] Shen L. M., Chen H. X. & Xiao F. & Wang S. W. The practical performance forecast and analysis of thermoelectric module from macro to micro. Energ. Convers Manage 100, 23–29 (2015).

[b13] Toberer E. S., Zevalkink A., Crisosto N. & Snyder G. J. The Zintl Compound Ca_5_Al_2_Sb_6_ for Low-Cost Thermoelectric Power Generation. Adv. Funct. Mater. 20, 4375–4380 (2010).

[b14] Zevalkink A., Toberer E. S., Bleith T., Flage-Larsen E. & Snyder G. J. Improved carrier concentration control in Zn-doped Ca_5_Al_2_Sb_6_. J. Appl. Phys. 110, 013721-1–013721-5 (2011).

[b15] Zevalkink A., Swallow J. & Snyder G. J. Thermoelectric Properties of Mn-Doped Ca_5_Al_2_Sb_6_. J. Electron. Mater. 5, 813–818 (2012).

[b16] Zevalkink A., Swallow J., Ohno S., Aydemir U. & Bux S. . Thermoelectric properties of the Ca5Al2-xInxSb6 solid solution. Dalton T. 43, 15872–15878 (2014). 10.1039/c4dt02206h 25226576

[b17] Yan Y. L. & Wang Y. X. Crystal structure, electronic structure, and thermoelectric properties of Ca_5_Al_2_Sb_6_. J. Mater. Chem. 21, 12497–12502 (2011).

[b18] Ye L. Y., Wang Y. X., Yang J. M., Yan Y. L. & Zhang J. H. . Electronic structure and thermoelectric properties of the Zintl compounds Sr_5_Al_2_Sb_6_ and Ca_5_Al_2_Sb_6_: first-principles study. RSC Adv. 5, 50720–50728 (2015).

[b19] Yang G., Cui H. T., Ma D. W. & He C. Z. The elastic and thermoelectric properties of the Zintl compound Ca5Al2Sb6 under high pressure. J. Appl. Phys. 116, 223709 (2014).

[b20] Ong K. P., Singh D. J. & Wu P. Analysis of the thermoelectric properties of n-type ZnO. Phys. Rev. B 83, 115110-1–115110-5 (2011).

[b21] Tran F. & Blaha P. Accurate band gaps of semiconductors and insulators with a semilocal exchange-correlation potential. Phys. Rev. Lett. 102, 226401-1–226401-4 (2009). 19658882 10.1103/PhysRevLett.102.226401

[b22] Snyder G. J. & Toberer E. S. Complex thermoelectric materials. Nature Mater. 7, 105–114 (2008). 18219332 10.1038/nmat2090

[b23] Zevalkink A., Pomrehn G. S., Johnson S., Swallow J. & Gibbs Z. M. . Influence of the Triel Elements (M = Al, Ga, In) on the Transport Properties of Ca_5_M_2_Sb_6_ Zintl Compounds. Chem. Mater. 24, 2091–2098 (2012).

[b24] Atakulov S. B., Zaynolobidinova S. M., Nabiev G. A., Nabiyev M. B. & Yuldashev A. A. Theory of transport phenomena in polycrystalline lead chalcogenide films. Mobility. Nondegenerate statistics. Semiconductors 47, 879–883 (2013).

[b25] Blöchl P. E. Projector augmented-wave method. Phys. Rev. B 50, 17953–17978 (1994). 10.1103/physrevb.50.17953 9976227

[b26] Kresse G. & Furthmller J. Efficient iterative schemes for ab initio total-energy calculations using a plane-wave basis set. Phys. Rev. B 54, 11169–11186 (1996). 10.1103/physrevb.54.11169 9984901

[b27] Kresse G. & Joubert D. From ultrasoft pseudopotentials to the projector augmented-wave method. Phys. Rev. B 59, 1758–1775 (1999).

[b28] Perdew J. P., Burke K. & Ernzerhof M. Generalized Gradient Approximation Made Simpl. Phys. Rev. Lett. 77, 3865–3868 (1996). 10062328 10.1103/PhysRevLett.77.3865

[b29] Monkhorst H. J. & Pack J. D. Special points for Brillouin-zone integrations. Phys. Rev. B 13, 5188–5192 (1976).

[b30] Blaha P., Schwarz K., Madsen G. K. H., Kvasnicka D. & Luitz J. WIEN2K. An Augmented Plane Wave + Local Orbitals Program for Calculating Crystal properties, Vienna University of Technology, Vienna, Austria (2001).

[b31] Becke A. D. Density‐functional thermochemistry. III. The role of exact exchange. J. Chem. Phys. 98, 5648–5652 (1993).

[b32] Engel E. & Vosko S. H. Exact exchange-only potentials and the virial relation as microscopic criteria for generalized gradient approximations. Phys. Rev. B 47, 13164–13174 (1993). 10.1103/physrevb.47.13164 10005620

[b33] Perdew J. P. & Wang Y. GENERALIZED GRADIENT APPROXIMATION (GGA) PW91. Phys. Rev. B 45, 13244–13249 (1992).

[b34] Jone W. & March N. H. Theoretical Solid State Physics, Courier Dover, New York (1985).

[b35] Madsen G. K. H., Schwarz K., Blaha P. & Singh D. J. Electronic structure and transport in type-I and type-VIII clathrates containing strontium, barium, and europium. Phys. Rev. B 68, 125212–125217 (2003).

