# Supplementary material for: Optimizing the Dopant and Carrier Concentration of Ca5Al2Sb6 for High Thermoelectric Efficiency
Source: Sci Rep. 2016 Jul 13;6:29550. doi: 10.1038/srep29550 (PMC4942768; doi:10.1038/srep29550)
Supplement: Supplementary Information [file srep29550-s1.pdf]

Supplementary Materials for

Optimizing the Dopant and Carrier Concentration of $Ca_5Al_2Sb_6$ for High Thermoelectric Efficiency

Yuli Yan[a,b], Guangbiao Zhang[a], Chao Wang[a], Chengxiao Peng[a], Peihong Zhang[b,c], Yuanxu Wang[a*], Wei Ren[b†]

[a] *Institute for Computational Materials Science, School of Physics and Electronics, Henan University, Kaifeng 75004, People's Republic of China;* [b]*Department of Physics, International Center for Quantum and Molecular Structures, and Materials Genome Institute, Shanghai University, Shanghai 200444, China;* [c]*Department of Physics, University at Buffalo, State University of New York, Buffalo, New York 14260, USA*

(Dated: May 28, 2016)

* E-mail: wangyx@henu.edu.cn

† E-mail: renwei@shu.edu.cn

I. FIGURE CAPTIONS

Fig. S1. Anisotropic transport properties as a function of the carrier concentration for n-type (left) and p-type (right) single crystal $Ca_5Al_2Sb_6$ for T=300, 500, 800, 1000, and 1200 K.

Fig. S2. Thermal conductivity of $Ca_5Al_2Sb_6$ from Ref.13, Ref.14, Ref.15, and Ref.16.

Fig. S3. Transport properties as a function of the carrier concentration for n-type (left) and p-type (right) doping $Ca_{40}Al_{16}Sb_{47}Ge$ for T=300, 500, 800, 1000, and 1200 K.

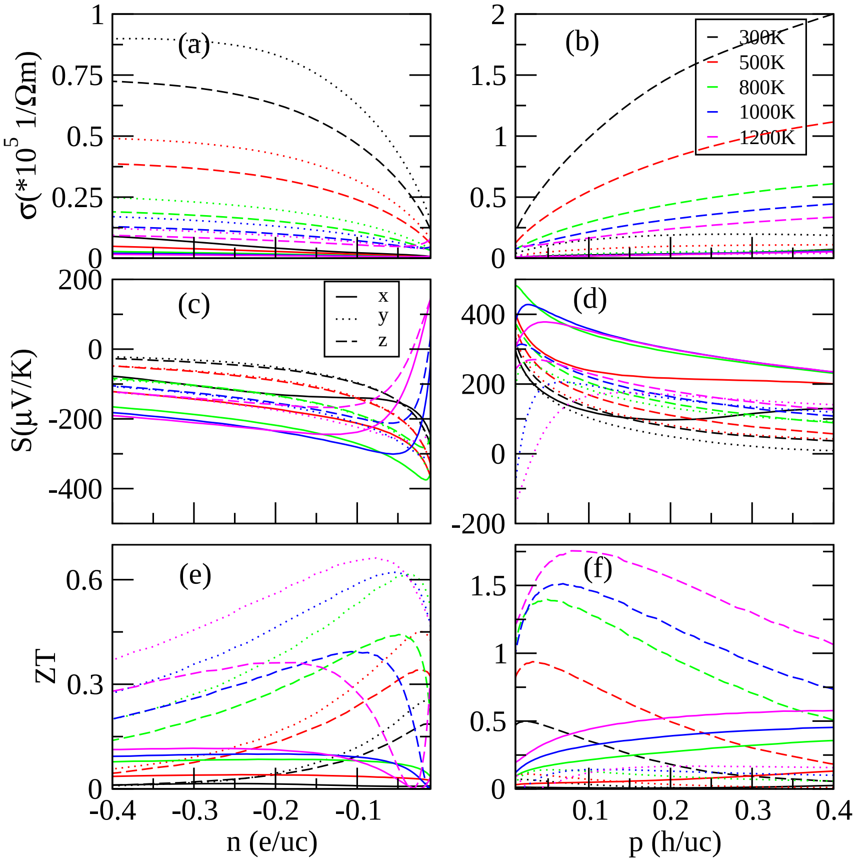

FIG. 1: Anisotropic transport properties as a function of the carrier concentration for n-type (left) and p-type (right) single crystal $Ca_5Al_2Sb_6$ for T=300, 500, 800, 1000, and 1200 K.

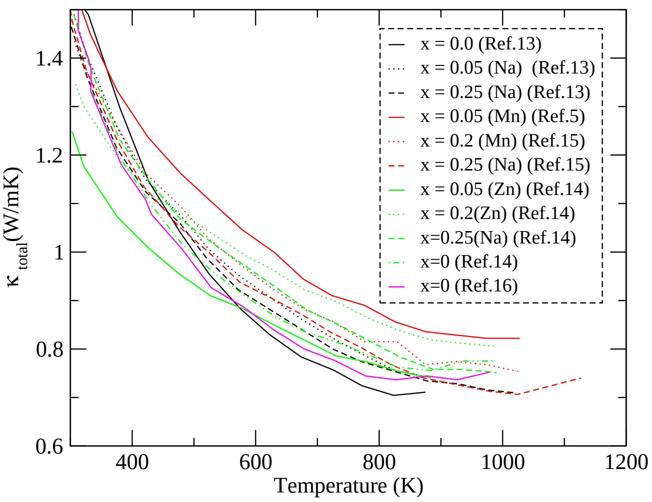

FIG. 2: Thermal conductivity of $Ca_5Al_2Sb_6$ from Ref.13, Ref.14, Ref.15, and Ref.16.

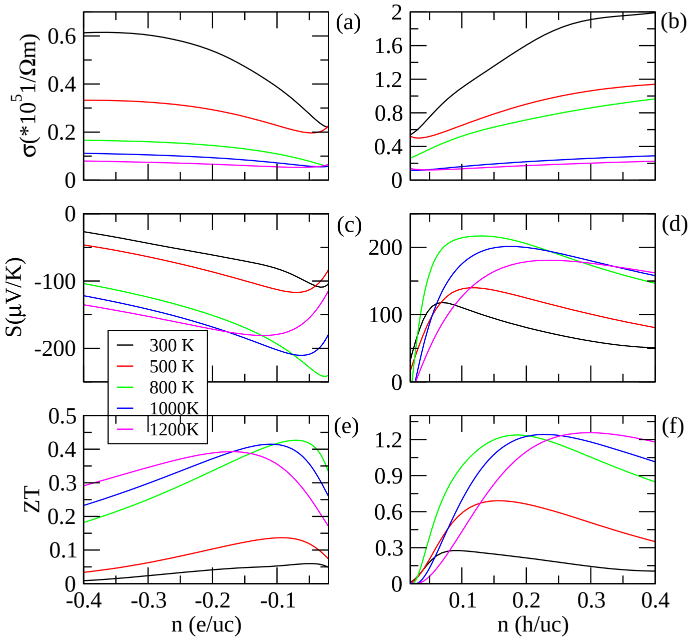

FIG. 3: Transport properties as a function of the carrier concentration for n-type (left) and p-type (right) doping $Ca_{40}Al_{16}Sb_{47}Ge$ for T=300, 500, 800, 1000, and 1200 K.
